# Do People Trust in Robot-Assisted Surgery? Evidence from Europe

**DOI:** 10.3390/ijerph182312519

**Published:** 2021-11-28

**Authors:** Joan Torrent-Sellens, Ana Isabel Jiménez-Zarco, Francesc Saigí-Rubió

**Affiliations:** 1 Faculty of Economics and Business Studies, Universitat Oberta de Catalunya (UOC), 08018 Barcelona, Spain; jtorrent@uoc.edu (J.T.-S.); ajimenezz@uoc.edu (A.I.J.-Z.); 2 Interdisciplinary Research Group on ICTs, 08018 Barcelona, Spain; 3 Faculty of Health Sciences, Universitat Oberta de Catalunya (UOC), 08018 Barcelona, Spain

**Keywords:** robot-assisted surgery (RAS), artificial intelligence (AI), technology acceptance model (TAM), logit regression, Europe

## Abstract

(1) Background: The goal of the paper was to establish the factors that influence how people feel about having a medical operation performed on them by a robot. (2) Methods: Data were obtained from a 2017 Flash Eurobarometer (number 460) of the European Commission with 27,901 citizens aged 15 years and over in the 28 countries of the European Union. Logistic regression (odds ratios, OR) to model the predictors of trust in robot-assisted surgery was calculated through motivational factors, using experience and sociodemographic independent variables. (3) Results: The results obtained indicate that, as the experience of using robots increases, the predictive coefficients related to information, attitude, and perception of robots become more negative. Furthermore, sociodemographic variables played an important predictive role. The effect of experience on trust in robots for surgical interventions was greater among men, people between 40 and 54 years old, and those with higher educational levels. (4) Conclusions: The results show that trust in robots goes beyond rational decision-making, since the final decision about whether it should be a robot that performs a complex procedure like a surgical intervention depends almost exclusively on the patient’s wishes.

## 1. Introduction

With advances in computing technology, artificial intelligence (AI) is becoming common, a higher-order family of applied knowledge capable of connecting lower-order technologies to generate innovations within economic and social systems [1,2]. AI devices can detect, capture, and analyse information and communicate data in real time, connecting with other technologies. Robots use AI to process and analyse data and to recognise and predict patterns. Indeed, AI could reshape medical care by improving both clinical and non-clinical applications [3,4,5,6,7,8,9,10]. In public health, AI could improve the early detection of sources of disease outbreaks [11], predict outcomes for critically ill patients, and predict adverse drug reactions [12].

In the healthcare sphere, different types of robots are used for a variety of purposes, including the early detection or treatment of a disease [13]; assistance for people with disabilities or cognitive issues to enable them to remain independent [14,15]; assistance for patients undergoing rehabilitation therapy [16]; the delivery of meals, medication and laundry in hospitals [17]; the provision of telemedicine services [18]; and the performance of surgery [19]. In the clinical sphere, robots are gradually being adopted to perform complicated operations, including minimally invasive surgery and guided non-surgical procedures. In a growing number of healthcare systems worldwide, robot-assisted surgery (RAS) is starting to be used. RAS is a minimally invasive technique capable of assisting surgeons with complicated surgical procedures [20,21,22,23].

Robots could absorb activities currently carried out by professionals [22,24], which would challenge traditional healthcare practices [23,25]. To what extent is it possible to foresee a near-future scenario in which minor routine surgery is directed by robots? And what are the patients’ or general public’s perceptions of having surgical procedures performed on them by robots, be it totally or partially? It is crucial to establish a robot strategy that is aligned with the objectives of the sector and its stakeholders. Without a patient-aligned strategy, any robot initiative is likely to remain at the pilot stages. So, knowing what the reasons are for people’s trust in or mistrust of robots being used in surgical interventions would represent a new contribution to the literature and would be useful for healthcare policy decision-making. 

The available evidence shows that there is a whole series of advantages associated with RAS. These advantages include reduced risks and errors in surgical interventions, shorter recovery times and lower financial costs [26,27,28,29,30,31]. The vast majority of the evidence has been provided by healthcare professionals and not by potential patients. The little available evidence provided by patients highlights changes in patient care systems and quality, and in the configuration of medical teams [32,33,34]. However, there is hardly any evidence relating to trust in RAS. Indeed, it is on this particular aspect that our study makes a new contribution to the literature. In our inquiry into the factors determining citizens’ trust in RAS, we expand the spectrum of assessments, we complement the results obtained from professionals, and we provide evidence to enable public policy to strengthen the presence of RAS in those areas where it deems it appropriate to do so.

Trust is an important variable in health-related decision-making, especially when it entails high risk and uncertainty. The aim of this article is to establish the factors that influence how people feel about having a medical operation performed on them by a robot. This study takes an integrated approach, considering drivers and barriers such as socioeconomic and cultural environments, sociodemographic factors, and psychographic indicators. To address this, we designed a predictive model and tested our research hypotheses for a representative sample of more than 26,500 European citizens.

## 2. Hypotheses and Model

In the specialised literature, there are numerous works analysing the factors that influence attitudes and intentions towards technology [35,36]. Among the various proposed models, the Technology Acceptance Model (TAM) is the theoretical proposal that is most widely applied to research into the acceptance of technology in the healthcare sphere [37]. Many works have shown that this model has the power to robustly explain variance in the intention to use, and use behaviour of, technology in general, and of e-health in particular [38,39,40]. However, the novelty of robot use in the healthcare sphere means that very few works have been done to explain the trust that citizens in general and patients in particular have in robots, taking into consideration the perceived usefulness and perceived ease of use thereof.

First, it should be noted that the usefulness of robotics in medicine is considerable. The perceived benefits of robot use have an influence on a patient’s trust in them. A body of literature suggests that RAS provides significant benefits. On the one hand, because of their ability to be programmed and their exact precision through stereoscopic vision in an area of interest, scalable movements, a wider range of axial movement [41] and the degree of computational accuracy [42], robots enable the specialist to better access risky areas or areas where there is no room for error. By doing so, RAS improves functional outcomes [26,27,28,29,30] and reduces morbidity rates for certain procedures [31,43]. Furthermore, RAS reduces the risk of surgery-related adverse events [43,44,45] by reducing operating times and technical errors, by improving access to areas of the body that are hard to reach, and by improving outcomes by eliminating (or minimising) the potential for human error, such as a surgeon’s tremors and vulnerability to fatigue [25], thereby helping the patient to recover faster and ensuring that the patient’s hospital stay is shorter [46,47,48]. This also leads to savings in the financial [27], time and psychological costs associated with the process [49,50,51].

On the other hand, a patient’s perception of robot use transforms the care received, reconfigures the work team [32,33], changes the quality of the care process [34], and has an influence on the patient’s trust in robots. Given the above, we consider that:

**Hypothesis** **1** **(H1).** *The individual’s perceived usefulness of robot use influences how he/she feels about having a medical operation performed by a robot.*

**Hypothesis** **1.1** **(H1.1).** *The perception that robots facilitate the performance of complex and dangerous tasks influences how people feel about having a medical operation performed by a robot.*

**Hypothesis** **1.2** **(H1.2).** *The perception that robots foster care innovation influences how people feel about having a medical operation performed by a robot.*

Secondly, the perceived ease of use of a robot largely depends on the training and experience of the professional using it. This perception is also influenced by the degree of relationship that the individual has with robots. A higher degree of relationship implies a higher degree of knowledge, and even of experience of use, thus enabling the individual to form an idea of the difficulty involved in handling them [52,53]. Given the above, we consider that:

**Hypothesis** **2** **(H2).** *The individual’s perceived ease of use of robots influences how people feel about having a medical operation performed by a robot.*

Thirdly, trust in having a medical operation performed by a robot may also be determined by the individual’s degree of knowledge of and emotional relationship with robots. This relationship is determined by the information available to him/her, and by his/her perception of and attitude towards them [54,55]. As noted by Hutchison, all of the above-mentioned elements are manifestations of the different dimensions of human behaviour: cognitive, affective, and behavioural [56]. Indeed, knowledge provides the basis for behaviour. Thus, even though a patient may not have any experience of robot use, he/she may know about its characteristics, usefulness, benefits, and risks. Similarly, knowledge provides the basis for the perception and opinion of, and even attitude towards, robots [57].

The basis of knowledge is information. The individual builds knowledge on the basis of information, and he/she establishes criteria on which to form his/her expectations and evaluative judgments [58]. The individual may resort to different sources to obtain infor-mation about robots, such as reports, scientific articles and/or popular science articles in physical or digital media. Another way of obtaining information is through specialised, regulated training in robots, in their usefulness and in how to use them [59].

Meanwhile, a knowledge of robots is the starting point from which individuals can form their perceptions of, and even attitudes towards, robots. Moreover, in the sphere of psy-chology, it is well documented that behaviour is strongly influenced by the psychological factors of perception and attitude [60]. Perception is defined as the apprehension of something through the senses or mind. Perception therefore relates to the basic senses (sight, taste, etc.) and to learning and experience. Conditioning and imitation are some of the non-cognitive learning mechanisms that predominate in an individual’s initial formation of his/her perceptions of robots. Consequently, factors associated with learning, motivation and context are some of the multi-layered aspects of consumer behaviour that go to form an individual’s perception. It should be noted that perception provides the basis for the individual’s opinions and, as noted by Köster and Mojet [61], these are, in turn, the basis on which subsequent behaviour is established.

On the other hand, attitude can be defined as an opinion or feeling about something or someone, or a way of behaving [62], though others define it as an individual’s thoughts on a given subject, based on his/her knowledge and assessment of it; an individual’s exposure to a topic may occur over a period of time, or his/her information may be obtained indirectly from others [63]. Hence, if the individual knows about and is in contact with robots, be it in a work-related or personal setting, it is to be expected that he/she will form a positive or negative attitude towards them. It is evident that, in general terms, an individual’s attitude towards robots is influenced to a considerable extent by the situation in which he/she perceives the risk and implication as being high. Surgical procedures are situations in which the individual is subject to a high level of tension, and the perceived risk is usually high [64]. Decision-making in situations like these is usually a complex process because of the individual’s high level of implication. Hence, it is to be expected that the individual’s attitude towards robots will have an influence on their use in procedures as complex as surgical operations. Given the above, we consider that:

**Hypothesis** **3** **(H3).** *The individual’s level of emotional relationship with robots influences how people feel about having a medical operation performed by a robot.*

**Hypothesis** **3.1** **(H3.1).** *The individual’s degree of knowledge of robots influences how people feel about having a medical operation performed by a robot.*

**Hypothesis** **3.2** **(H3.2).** *The individual’s perception of robots’ ability to perform his/her habitual work influences how people feel about having a medical operation performed by a robot.*

**Hypothesis** **3.3** **(H3.3).** *The individual’s attitude towards robots influences how people feel about having a medical operation performed by a robot.*

Fourthly, the individual’s sociodemographic characteristics may also determine how robots are perceived and evaluated and may have an influence on his/her attitude towards them. These characteristics not only condition his/her educational level or his/her degree of access to technology at personal and professional levels but may also affect matters as important as the choice of hospital where the operation will be performed [65]. In this respect, it has been found that patients undergoing robotic surgery are more likely to live in large metropolitan areas and have higher school graduation rates and incomes [66]. In addition, these characteristics are decisive when it comes to defining cultural and social aspects that are crucial to making a decision with a high level of implication, such as having a medical operation performed by a robot. Given the above, we consider that:

**Hypothesis** **4** **(H4).** *The individual’s sociodemographic characteristics influence how people feel about having a medical operation performed by a robot.*

**Hypothesis** **4.1** **(H4.1).** *The individual’s sociodemographic profile influences how people feel about having a medical operation performed by a robot.*

**Hypothesis** **4.2** **(H4.2).** *The individual’s place of residence influences how people feel about having a medical operation performed by a robot.*

Lastly, it should be noted that the individuals’ degree of experience of use determines his/her perception of and relationship with technology in general and robots in particular. Experience of use is the main source of information that the individual has available to him/her, on the basis of which he/she builds his/her knowledge and forms his/her perceptions [67] and expectations [68]. An individual’s experience of robot use determines perceptions of usefulness and ease of use of, and emotional relationship (knowledge, perception and attitude) with robots [69].

**Hypothesis** **5** **(H5).** *The individual’s prior experience of robot use has an influence on his/her perception of and relationship with robots.*

## 3. Materials and Methods

### 3.1. Study Design and Sample Selection

In order to obtain a representative sample and to analyse attitudes towards the impact of digitisation and automation on the daily lives of Europeans, the European Commission (2017) dedicated a Flash Eurobarometer (number 460) to a survey on the impact and use of digital technologies, digital skills, attitudes towards robotics, and digital health and care. Flash Eurobarometer is an ad-hoc statistical operation consisting of short, landline and mobile, telephone interviews on a topic of interest. Flash Eurobarometer 460 obtained data from a sample of 27,901 citizens aged 15 years and over in the 28 countries of the European Union. Approximately 1000 interviews per country were conducted in Belgium, Bulgaria, the Czech Republic, Denmark, Germany, Estonia, Ireland, Greece, Spain, France, Croatia, Cyprus, Latvia, Luxembourg, Hungary, the Netherlands, Austria, Poland, Portugal, Romania, Slovenia, Slovakia, Finland, Sweden, and the United Kingdom, whereas around 500 interviews per country were conducted in Italy, Lithuania, and Malta. The universe of the survey consisted of the 412,630,644 European Union citizens aged 15 years and over. The sample design for each country was probabilistic and representative. The margins of error at the 95% confidence level in the case of maximum indetermination (p = q = 50) were ±0.2% for the entire sample, and around ±1.4% for individual country samples, except for Italy, Lithuania, and Malta (±1.9%). The fieldwork was carried out on 18 and 23 March 2017. After analysing the frequencies and normality of the data, our resulting sample for analysis consisted of 26,592 European citizens.

As a research project promoted and funded by the European Commission, the usual ethical criteria applicable to social sciences and behavioural research were observed to obtain the data. Technical and ethical information about the questionnaire and the fieldwork can be found in the technical annexes to the final Eurobarometer 460 report [70].

### 3.2. Study Variables and Measurement Scale Construction

The model proposed in the previous section indicates how different variables have a direct influence on how people feel about having a medical operation performed by a robot. So, in order to test the hypotheses proposed in the model, it was necessary to measure each of the above-mentioned constructs using different variables, with feelings about having a medical operation performed by a robot being the variable to be explained. Table 1 shows the variables used in the study.

Appendix A shows the values of the descriptive statistics for the variables analysed.

In order to test hypothesis 1, the perceived usefulness of robots was measured using two dimensions, in terms of (a) facilitating the performance of complex and dangerous tasks and (b) influencing the evolution of and innovation in health services.

Both dimensions were obtained by performing exploratory factor analysis (EFA). This technique is used to analyse interrelations among a large number of variables and to explain these variables in terms of their common underlying dimensions. Table 2 shows all the statistical information relating to the analysis. All the variables of the correlation matrix showed high correlations and their determinant offered a value of 0.46. The Kaiser-Meyer-Olkin index value was 0.510 and the Bartlett’s test of sphericity value was 21,469.781 with a significance of 0.000. This analysis explained 78.5% of the variance. Moreover, the Cronbach’s alpha values confirmed the reliability of the scales. Additionally, the content and construct scales’ discriminant, convergent and nomological validity were addressed. With regard to the content, the scales were developed following a major review of the literature.

Regarding hypothesis 2 concerning ease of use, the variable was created from a set of original variables measuring an individual’s technology-related skills. An additive model was used for that purpose, in which all variables had the same weight. The variables considered in the model showed how the individual considered him/herself to be sufficiently skilled in the use of the technologies to do his/her job, to find a job, to use online public services, and to benefit from digital and online learning opportunities.

## 4. Results

### 4.1. Descriptive Analysis

The sample consisted of a total of 26,592 individuals. Of these, 45.1% were men and 54.9% were women. By age, 8.9% were 24 years old or under, whereas 20.5% were between 25 and 39 years old, 24.2% were between 40 and 54 years old, and more than 46.4% were 55 years old or over. Regarding education, 56.5% stated that they had higher education qualifications, since they had studied for nearly 20 years. In terms of civil status, 64% lived with a partner (mainly married couples with children), but it is important to underscore that a high number of people lived alone, either because they were single (17%) or widowed (10%). Lastly, in relation to social class, it should be noted that a significant percentage of the total number of individuals in the sample (46.3%) was middle class, though 26.4% of those surveyed described themselves as working class.

Regarding place of residence, 39.5% lived in medium-sized urban areas, although it is important to point out that 32.7% stated that they lived in rural areas. We could conclude that the population consisted principally of upper-middle-aged women (over 40 years old) with a high educational level, who lived in households consisting of several members and resided in medium-sized urban areas.

Their degree of knowledge and experience of robots could be deemed low. Of the total, 47% stated that they had read about robots, but only 5.3% indicated that they had used them at work, and just 7.5% had done so at home. Nevertheless, a high percentage of people (22.6%) believed that robots would be able to partially or fully do the work that they, as individuals, habitually did.

### 4.2. How He/She Feels about Having a Medical Operation Performed on Him/Her by a Robot

In order to test each variable’s capacity to influence an individual’s trust in using robots to perform operations, a logistic analysis of variance was performed (Table 3).

The model obtained was significant, explaining 16.6% of variance. Of the analysed variables, attitude towards robots (B = −0.425) and perceived benefits in relation to both performance of professional activity and care innovation (B = −0.385 and B = −0.305, respectively) had the highest explanatory power. After these, in descending order of importance, were information about robots (B = −0.289) and perception of robots (B = −0.222). Lastly, it should be noted that other variables such as experience of robot use (B = 0.121) or those relating to sociodemographic characteristics such as the individual’s gender (B = −0.372), age (B = 0.150) and social class (B = 0.106) had considerable explanatory power for the level of trust in robots.

It is worth pointing out that the variables with higher explanatory power had an inverse relationship with the independent variable. Thus, it was considered that, as the attitude towards and perceived benefits and knowledge of robots increased, trust in their use when performing an operation tended to decrease.

#### 4.2.1. Perceived Usefulness of Robots in Relation to Experience of Robot Use

Experience of robot use had a positive effect on trust. In order to establish how the different levels of experience of use would affect the individuals’ perception of and relationship with robots, and, ultimately, their trust in having an operation performed on them by robots, the whole sample was divided into three large groups by degree of experience: (1) those with no experience; (2) those with average experience; and (3) those with considerable experience. Regarding level of use, 88.3% did not use robots at all, 10.5% used them to an average extent and just 1.2% to a high extent.

Table 4 shows the results obtained from the test. As can be observed, the three models obtained were significant, explaining between 15.2% and 24.8% of variance.

When an individual did not have experience of robot use, the model variable with the highest explanatory power was attitude towards robots (B = −0.413). After that, in descending order of importance, were perceived benefits of robot use, in relation to both care innovation and facilitating the performance of professional activity, with values of B = −0.360 and B = −0.310, respectively, and lastly, information about robots (B = −0.251) and perception of robots (B = −0.244). Worth noting is the negative relationship between the different variables and trust.

Thus, again, it was considered that, as attitude towards and perceived benefits and knowledge of robots increased, trust in their use when performing an operation tended to decrease.

Meanwhile, for this group of individuals, the sociodemographic variables gender and age also had explanatory power for the trust individuals had in robots, with coefficient values of B = −0.383 and B = 0.143, respectively.

When the individuals’ level of robot use was high, their trust was strongly affected by the perceived benefits in relation to employment, by the information available to them, and by their attitude towards robots (B = −0.538, B = −0.506, and B = −0.502, respectively). After these, in descending order of importance, were sociodemographic variables such as age and educational level, with coefficients of B = −0.207 and B = 0.179, respectively.

Lastly, when the individuals’ experience of use was high, the variables having a greater influence on trust were information available to them about robots (B = −0.712), attitude towards robots (B = −0.576), perception of robots (B = −0.448), and perception of robots facilitating the performance of certain tasks (B = −0.424). It should be noted, however, that out of the sociodemographic variables, the only one that had a significant effect was gender, with a very high coefficient of B = −0.946.

Appendix B shows the hypotheses and their degree of fulfilment.

#### 4.2.2. Perceived Usefulness of Robots in Relation to Sociodemographic Variables

In order to analyse the effects of sociodemographic variables on trust in robots, the previous model was re-run, having first split the population by gender, age and educational level.

Regarding gender, we found that 51.8% of the sample was male and 45.1% female (Table 5). For men, the variable with the highest explanatory power was attitude towards robots (B = −0.427), followed by information (B = −0.412) and benefits obtained (B = −0.383 and B = −0.284). For women, the most relevant variables were attitude towards robots (B = −0.435) and perceived benefits (B = −0.380 and B = −0.380). The negative relationship between the different variables and trust was apparent in both groups. However, it should also be noted that, unlike men, women placed greater importance on the availability of secondary information about robots (B = −0.294). Meanwhile, it is also worth noting that prior experience of robot use was a relevant variable in terms of men having trust in robots (B = 0.159), whereas the relationship between the two variables was not significant for women.

Regarding age, we found four distinct groups: individuals between 15 and 24 years old (4.7%), between 25 and 39 years old (32.8%), between 40 and 54 years old (39.8%) and 55 years old or over (22.5%) (Table 5). For all age groups, variables relating to the perception of, attitude towards and availability of secondary information about robots were important and had a significant negative impact on trust. Similarly, the perceived benefits variable was relevant in all cases, also with a significant negative relationship.

It should be noted that, for the youngest group, the availability of information was crucial (B =−0.663). This was followed by perceived benefits (B = −0.539 and B = −0.530). For the 25–39-year-old group, the most important variable was attitude towards robots (B = −0.527), followed by their usefulness for innovation in work-related activity (B = −0.385). For the 40–54-year-old group, the most relevant variables were perceived usefulness in relation to the robots’ innovation capacity (B =−0.408) and attitude towards robots (B = −0.365). Lastly, for the 55-year-old or over group, the most relevant variables were attitude towards robots (B = −0.514) and availability of secondary information about robots (B = −0.510).

Worthy of note is the fact that the experience of robot use variable only showed a significant positive result for the 40–54-year-old group (B = 0.226). The variable was not significant in the other groups.

Finally, we found that the population had a high educational level. Indeed, in terms of the number of years of education, 6.2% of the individuals stated they had fewer than 15, 47.1% had between 16 and 19, and 46.7% had more than 20.

Table 5 shows the results for the three logits. They all display explanatory power, with a Nagelkerke’s R-squared coefficient ranging between 11.1% and 15.9%.

It is interesting to note that, for the group with the fewest years of education, the only relevant variables were the usefulness of robots for performing simple tasks (B = −0.438) and ease of use (B = 0.401). Meanwhile, for those groups with higher educational levels, it was found that the most relevant variables were attitude towards robots (B = −0.274 and B = −0.477), their usefulness for innovation in work-related activity (B = −0.317 and B = −0.438), and usefulness in relation to robots’ capacity to perform tasks (B = −0.248 and B = −0.347).

Lastly, it should be noted that the experience of robot use variable only showed a significant positive effect for the group of individuals with 16 to 19 years of education (B = 0.168). The variables did not display significant values for the other groups. Appendix C shows the degree of fulfilment of the hypotheses for the gender, age, and educational level variables.

## 5. Discussion

### 5.1. Research Contributions

By focusing our study on the analysis of trust that citizens (patients or future patients) have in RAS, we wanted to expand the scope of analysis to include a group of stakeholders that is not always taken into consideration when planning RAS strategies or public policies. In fact, and adding to the little available evidence from the user or patient perspective [32,33,34], our study is about establishing the factors that predict European citizens’ trust in RAS. Our ultimate intention has been to provide additional evidence so that public decision-makers or strategy designers can balance professionals’ positive perceptions against citizens’ reticence.

Based on the analysis of a large representative sample consisting of more than 27,901 citizens aged 15 years and over from 28 European countries in 2017, a model comprising the motivational, sociodemographic, and experience factors that predict trust in RAS was designed and tested. In general, the results obtained indicate that, as the experience of using robots increased, the predictive coefficients related to information, attitude and perception of robots became more negative. Furthermore, sociodemographic variables played an important predictive role. The effect of experience on trust in RAS was greater among men, people between 40 and 54 years old, and those with higher educational levels.

Health robotic could effectively perform tasks such as taking people’s temperature in public areas or at ports of entry, providing quarantined patients with support, and enabling virtual care. They could also be used to carry out many of the tasks deemed thankless, dirty or dangerous during the pandemic, such as decontamination, waste delivery and handling, or monitoring quarantine compliance [71,72]. Within this context, the majority of studies into the effects of RAS are based on analyses of healthcare professionals’ assessments, which generally indicate positive effects on surgical intervention risk reduction, efficiency, and quality, and on the minimisation and subsequent recovery of costs linked to such interventions [26,27,28,29,30,31].

However, the definitive implementation of robots in the healthcare sphere, with all the opportunities they offer and all the challenges they pose, will almost certainly result in the need to undertake a complete strategic overhaul of health services. Many obstacles still need to be overcome before the potential of robotics can be unleashed. One such obstacle is, without doubt, patients’ trust. It is known that patients’ trust is an important determinant of behaviours and experiences in both medical care and the doctor–patient relationship [73]. However, given its importance in surgical procedures, establishing trust should be a priority when faced with the possibility of new technologies such as RAS being integrated into surgical procedures. That is why it is important to understand how the characteristics of robotics affect patient’s trust, and what influences and leads to humans’ trust in robots when faced with the possibility of being operated on, autonomously, by a robot.

A patient’s intention and decision to have surgical procedures performed on him/her by robots, be it totally or partially, entail considerable implication and a high level of perceived risk on his/her part. Moreover, their adoption requires a longer process and more time. Once it has been understood how the characteristics of robotics influence a patient’s trust, it is then necessary to understand how important the dimensions of his/her trust are to the use of and support by robotics in the surgical sphere. Patients’ trust is not a singular, generalised phenomenon, but rather a series of nuanced relationships based on specific behaviours and expectations. Previous studies on robots being used in older people’s health management [74] or by service providers [75] have noted ambiguities in the definition of factors contributing to the establishment of trust, as well as the complexity of empirically isolating these factors [76].

Experience of robot use has a positive effect on trust, as do more positive attitudes towards robots (by increasing the degree of knowledge about their characteristics and benefits) [77]. However, if the focus were to be placed on the clinical setting and, in particular, on RAS, then prior expectations might lead to more negative feelings towards robots. Indeed, in our research, we contrasted the negative relationship between the majority of the predictors of ease of use, expected benefits, and information about, perception of, and attitude towards robots with trust in the use of robotics in a surgical intervention. In fact, the only non-sociodemographic predictive variable that seemed to have a positive relationship with trust in robots was prior experience of robot use. In other words, whereas all the motivational predictors relating to ex-ante information about robots had negative predictive power for trust, only ex-post experience, i.e., having previously used robots, generated trust. This important motivational limitation, which confines trust in robots solely to prior robot use in other spheres, is almost certainly due to the fact that the association between robots and the operating theatre is perceived as an extremely novel use of technology with potential risk or a very considerable need for cultural change. In fact, in research on predictors of use of all types of digital technologies, similar results can be found in perceived uses of such technologies in their early or preliminary stages [40,78,79,80].

Having identified the importance of prior experience of robot use, we analysed the predictors of trust for three different levels of experience (zero use, average use, and high use). The results indicated a clear substitution effect between ex-post experience and ex-ante perceptions. That is, as experience of robot use increased, the predictive coefficients relating to information about, attitude towards and perception of robots became more negative, as did the one relating to robots facilitating the performance of tasks. In other words, as prior experience of robot use increased, the more negative the effects of predictors linked to information about, attitudes towards and ex-ante perceptions of robot use were. This result, combined with the previous one, suggests that experience had a dual effect on trust. The first effect, or level effect, determined that prior experience of robot use was decisive for motivating trust in surgical interventions performed totally or partially by robots. The second effect, or marginal effect, determined that the greater the prior experience of robot use, the bigger the negative effects of predictors not linked to ex-ante experience. Experience of use generated trust, and at the same time, greater experience generated more mistrust of prior perceptions not linked to use.

The results of our research also determined that variables of a sociodemographic nature played an important predictive role. The results obtained for gender, age and educational level are particularly interesting. We performed a detailed analysis in all three cases. Regarding gender, for men, we found a higher positive incidence of experience, as well as higher predictive power (mistrust) of non-experiential variables linked to information about robots. For women, mistrust was based on a greater preponderance of perceptions and the anticipated facilitation of the performance of tasks. Although age usually leads to positive feelings towards robots [81], our results showed that, as age and, ultimately, experience of robots increased, age only had a significant positive impact on trust in the 40- to 54-year-old group. Meanwhile, the mistrust of non-experiential variables, especially those relating to information, perceptions and facilitation, reduced with age. Lastly, the analysis of educational level (years of completed education) also produced some interesting results. Firstly, we found that the predictive power of experience for trust in robots for surgical interventions increased with more years of education. Secondly, we found that the behaviour of experiential variables was more erratic. In short, we confirmed that the effect of experience on trust in robots for surgical interventions was higher among men, individuals aged between 40 and 54, and those who had higher educational levels. This sociodemographic characterisation could also be useful for the implementation of support policies for the robotisation of the health system.

### 5.2. Practical Implications

From the viewpoint of healthcare management and policy, our results suggest that the incentivisation of RAS should consider different motivational routes. To overcome the strong resistance to the implantation of robots in surgical interventions, it is highly recommended to take advantage of the positive synergies that prior use of this technology produces in spheres other than that of healthcare. The use of positive perceptions of surgical robotics held by strata of the population that already use robotics in their places of work or in the domestic setting is a good starting point for improving the situation. Meanwhile, healthcare management and policy could work on the entire set of negative perceptions of robotics held generally by the population that has never come into contact with robots. It is particularly important to consider the social implantation phase of the use of robotics in surgical practice, especially as citizens may see this technology as being in its early stages and risky, and as one that poses major cultural challenges.

So, despite the considerable—and more than proven—benefits that robot use can bring to a patient when performing a surgical intervention, it should be borne in mind that, when it comes to health, the patient is not entirely rational. The decision to have an operation usually entails high risk and uncertainty for the patient because it implies that he/she is placing his/her most precious ‘asset’ in the hands of a third party, without any indication—or guarantee—of what the outcome will be like. If, in addition, the operation is performed by an autonomous robot, i.e., without the surgeon’s assistance, the level of risk and uncertainty will increase, thus leading to a rise in stress levels. By parameterising the reasons that generate trust in and mistrust of robots, mainly by highlighting experience of use as a key element for generating trust, our research makes a new contribution to the state of the art and draws practical implications of robot use for healthcare policy and practice.

Beyond the importance of experience, the analysis of non-experiential motivations suggests that the availability of more and better information on the surgical procedure and on potential health outcomes will have a decisive impact on the patient’s trust and, ultimately, on the decision taken by him/her in this regard. On some occasions, this information can be obtained from indicators that provide evidence of the potential outcome, whereas on others, it can be obtained directly from the patient via research into his/her motivations. In our study, we found that some sociodemographic characterisations were more inclined towards trust in robot use for surgical practices. As the successes of robotics in medicine become more evident, it may require governments and funders to formulate distinct strategies aimed at groups that are more likely to trust in robots. However, given that the effect of experience on trust is twofold, i.e., first there is a level effect (greater experience of use equals more trust) and then a marginal effect (greater experience equals more mistrust of non-experiential motivations), it is important for public policy to take both aspects into account. To promote the level effect, research into this area needs increased funding, on the one hand to address regulatory, ethical, and legal issues, and, above all, the issue of liability. On the other, it is vital to produce more scientific evidence of the clinical efficacy and viability of this technology. Its standardisation may favour the spread of surgical skills in developing countries, via the Internet or via mobile platforms using telemedicine solutions that are well-controlled by AI-based algorithms [82]. Within the Horizon Europe research and innovation programme, the European Commission intends to create a new public–private partnership to join forces and ensure the coordination of AI, data, and robotics research and innovation (Action 5) [71].

According to Wehner et al. [83], it is a rule for a robot not to harm humans or allow humans to be harmed. Faced with a potential scenario in which the general public accepts that robotics will take potentially critical decisions [42], and in which the evolution of technology will lead to a reduction in production costs [84], the penetration of robotics in the surgical sphere could optimise the outcomes of and increase access to surgical care [79], as well as democratise surgical care and standardise surgical outcomes regardless of economic and geographical restraints [82,85].

### 5.3. Limitations

This study has a series of limitations that need to be considered. First, the data for this analysis came from a cross-sectional sample compiled in 2017. The expanded use of robotics since then, both in general and in the surgical sphere in particular, may have changed the patients’ experiences and perceptions of RAS. Furthermore, qualitative research would allow a more in-depth understanding to be had of how the different dimensions of trust influence patients’ behaviour and expectations in terms of their trust in having an operation performed on them by robots, be it totally or partially.

## 6. Conclusions

In any human–robot relationship, trust is important because it directly affects people’s dispositions towards accepting the information that robots produce and following the suggestions they make [86]. If, moreover, we focus on the clinical setting and particularly the surgical sphere, the effective remote implementation of robots should take into account those factors that have an influence on the general public’s trust. Based on a large population sample in Europe, this study found a broad set of misgivings about undergoing RAS due to a lack of trust in it, thus providing new evidence to the debate on the acceptance of RAS by European citizens. In fact, only two variables, namely previous experience of robot use and perceived ease of use of robots, were capable of predicting trust in RAS. In addition, the motivations for mistrust (information about, attitude towards and perception of robots) grew with experience of their use. These findings have clear implications for the design of RAS health strategies and policies. Indeed, faced with a care model based on close collaboration between the professional and the patient, the final decision on RAS depends almost exclusively on the wishes of the patient. These wishes are clearly related to trust. Thus, RAS implantation strategies and policies must consider the factors that hinder or promote patient trust.

## Figures and Tables

**Table 1:** Model variables.

**Feels about having a medical operation performed by a robot**		The individual’s trust in being operated on by a robot. Dichotomous variable: 0 = negative; 1 = positive.
**Ease of use of robots**		Metric variable indicating the individual’s perceived ease of use of robots.
**Benefits derived from robot use**	**Benefits in performing the work**	Metric variable indicating how the individual rates the benefits in performing the work.
	**Affects employment**	Metric variable indicating the degree to which the individual considers that robot use affects the way he/she receives care.
**Information about robots**		Dichotomous variable indicating whether, in the last 12 months, the individual has heard, read or seen anything about robots.
**Perception of robots**		Variable measured on a 5-point Likert scale indicating the individual’s perception of how easy it is for a robot to perform his/her current work.
**Attitude towards robots**		Variable measured on a 5-point Likert scale indicating the individual’s attitude towards robots.
**Experience of robot use**		Categorical variable indicating whether the individual has experience of robot use, be it in a professional or domestic setting: 0 = no experience; 1 = average experience; 2 = considerable experience.
**Gender**		Dichotomous variable indicating the individual’s gender: 1 = Male; 2 = Female.
**Age**		Categorical variable indicating the subject’s age range (in years): 1 = 15–24; 2 = 25–39; 3 = 40–54; 4 = 55 or over.
**Family situation**		Categorical variable indicating the individual’s civil status: 1 = Single, without children; 2 = Single, with children; 3 = Married or in a partnership, without children; 4 = Married or in a partnership, with children.
**Occupation**		Categorical variable indicating the individual’s occupation: 1 = Self-employed; 2 = Manager; 3 = Other white collar; 4 = Manual worker; 5 = Homemaker; 6 = Unemployed; 7 = Retired; 8 = Student.
**Educational level**		Categorical variable indicating the individual’s educational level: 1 = More than 15 years; 2 = 16-19 years; 3 = 20 years; 4 = Still in education; 5 = Part-time education.
**Social class**		Categorical variable indicating the social class to which the individual consider he/she belongs: 1 = Working class; 2 = Lower middle class; 3 = Upper middle class; 4 = Upper class; 5 = Upper upper class.
**Type of community where he/she lives**		Categorical variable indicating the type of area in which he/she lives: 1 = Rural area or village; 2 = Medium-sized town/city; 3 = Big town/city.

**Table 2:** Factor analysis results.

	Has an Influence on Employment	Facilitates Activities
**Fosters innovation**	0.900	
**Does not destroy jobs**	0.893	
**Helps to perform tasks**		0.877
**Allows dangerous activities to be carried out**		0.863
**Eigenvalue**	1.622	1.519
**% variance explained**	40.540	37.960
**Cronbach’s alpha**	0.760	0.669

**Table 3:** Results of logistic regression for the sample as a whole.

	Model Variable	Beta	SE	Wald	df	Sig.	Exp(B)
d15a (occupation)	**Occupation**	−0.035	0.008	17.558	1	0.000	0.965
d10 (gender)	**Gender**	−0.372	0.049	57.575	1	0.000	0.689
d8r2 (education)	**Educational level**	0.106	0.027	15.222	1	0.000	1.112
d25 (community type)	**Type of community where he/she lives**	0.025	0.031	0.638	1	0.424	1.025
d40a (household composit.)	**Family situation**	0.002	0.024	0.006	1	0.936	1.002
d63 (social class)	**Social class**	0.061	0.019	10.170	1	0.001	1.062
d11r1 (age)	**Age**	0.150	0.030	25.517	1	0.000	1.162
Qd8 (experience)	**Experience of robot use**	0.121	0.052	5.441	1	0.020	1.128
qd9 (I read about robots)	**Information about robots**	−0.289	0.052	31.208	1	0.000	0.749
qd10 (attitude towards robots)	**Attitude towards robots**	−0.425	0.036	137.677	1	0.000	0.654
qd11 (robot perception)	**Perception of robots**	−0.222	0.027	69.257	1	0.000	0.801
QD123 (ease of use new)	**Ease of use of robots**	0.075	0.038	4.010	1	0.045	1.078
BENF2	**Fosters innovation**	−0.385	0.039	97.617	1	0.000	0.680
BENF1	**Facilitates the performance of tasks**	−0.305	0.027	130.317	1	0.000	0.737
Constant		0.163	0.298	0.298	1	.585	1.177
**Hosmer-Lemeshow chi-square**		34.648 (0.000)					
**Nagelkerke’s R-squared**		0.166					

**Table 4:** Logit model results, by level of use.

Model Variable	Zero Use		Average Use		High Use	
	Beta	Sig.	Beta	Sig.	Beta	Sig.
**Occupation**	−0.041	0.000	−0.009	0.667	0.020	0.728
**Gender**	−0.383	0.000	−0.207	0.089	−0.946	0.003
**Educational level**	0.098	0.002	0.179	0.003	−0.027	0.853
**Type of community where he/she lives**	0.017	0.635	0.085	0.251	−0.067	0.731
**Family situation**	−0.003	0.912	−0.005	0.923	0.036	0.779
**Social class**	0.061	0.003	0.063	0.224	−0.044	0.757
**Age**	0.143	0.000	0.222	0.002	0.068	0.740
**Information about robots**	−0.251	0.000	−0.506	0.001	−0.712	0.053
**Attitude towards robots**	−0.413	0.000	−0.502	0.000	−0.576	0.037
**Perception of robots**	−0.244	0.000	−0.108	0.082	−0.448	0.009
**Ease of use of robots**	0.088	0.037	−0.030	0.748	0.393	0.130
**Fosters innovation**	−0.360	0.000	−0.538	0.000	−0.201	0.501
**Facilitates the performance of tasks**	−0.310	0.000	−0.279	0.000	−0.424	0.018
**Hosmer-Lemeshow chi-square**	20.745 0.008		13.976 0.082		12.534 0.129	
**Nagelkerke’s R-squared**	0.152		0.174		0.248	

**Table 5:** Logit results model, by gender, age and educational level of the individuals.

Logit Model Results, by Gender									
	Model Variable	Male				Female			
		Beta	Sig.			Beta			Sig.
Qd8 (experience)	**Experience of robot use**	0.159	0.016			0.109			0.183
qd9 (I read about robots)	**Information about robots**	−0.412	0.000			−0.294			0.000
qd10 (attitude towards robots)	**Attitude towards robots**	−0.427	0.000			−0.435			0.000
qd11 (robot perception)	**Perception of robots**	−0.153	0.000			−0.239			0.000
QD123 (ease of use new)	**Ease of use of robots**	0.100	0.045			0.068			0.221
BENF2	**Fosters innovation**	−0.383	0.000			−0.380			0.000
BENF1	**Facilitates the performance of tasks**	−0.294	0.000			−0.380			0.000
Constant		0.073	0.797			−0.016			0.961
	**Hosmer-Lemeshow chi-square**	36.039 (0.000)				14.099 (0.079)			
	**Nagelkerke’s ** **R-squared**	0.151				0.13.5			
**Logit results, by age of the individuals**									
	**Model variable**	**15 to 24**	**25 to 39**			**40 to 54**		**55 and over**	
		**Beta**	**Sig.**	**Beta**	**Sig.**	**Beta**	**Sig.**	**Beta**	**Sig.**
Qd8 (experience)	**Experience of robot use**	0.224	0.383	0.100	0.250	0.226	0.004	0.144	0.221
qd9 (I read about robots)	**Information about robots**	−0.663	0.020	−0.282	0.002	−0.353	0.000	−0.510	0.000
qd10 (attitude towards robots)	**Attitude towards robots**	−0.398	0.039	−0.527	0.000	−0.365	0.000	−0.514	0.000
qd11 (robot perception)	**Perception of robots**	−0.531	0.000	−0.205	0.000	−0.202	0.000	−0.175	0.002
QD123 (ease of use new)	**Ease of use of robots**	−0.112	0.521	0.025	0.710	0.028	0.628	0.261	0.001
BENF2	**Fosters innovation**	−0.539	0.013	−0.385	0.000	−0.408	0.000	−0.288	0.000
BENF1	**Facilitates the performance of tasks**	−0.530	0.001	−0.300	0.000	−0.298	0.000	−0.408	0.000
Constant		1.704	0.088	0.351	0.342	0.176	0.598	−0.260	0.568
	**Hosmer-Lemeshow chi-square**	10.241 (0.249)		12.651 (0.124)		18.839 (0.016)		13.685 (0.090)	
	**Nagelkerke’s R-squared**	0.212		0.141		0.134		0.194	
**Logit results, by educational level**									
	**Model variable**		**≤15 years of education**		**16–19 years of education**			**+20 years of education**	
			**Beta**	**Sig.**	**Beta**	**Sig.**		**Beta**	**Sig.**
Qd8 (experience)	**Experience of robot use**		0.167	0.619	0.168	0.053		0.108	0.100
qd9 (I read about robots)	**Information about robots**		−0.323	0.237	−0.363	0.000		−0.274	0.000
qd10 (attitude towards robots)	**Attitude towards robots**		−0.654	0.001	−0.377	0.000		−0.477	0.000
qd11 (robot perception)	**Perception of robots**		−0.206	0.124	−0.219	0.000		−0.200	0.000
QD123 (ease of use new)	**Ease of use of robots**		0.401	0.063	0.087	0.132		0.049	0.349
BENF2	**Fosters innovation**		−0.139	0.472	−0.317	0.000		−0.438	0.000
BENF1	**Facilitates the performance of tasks**		−0.438	0.004	−0.248	0.000		−0.367	0.000
Constant			−1.270	0.285	−0.090	0.781		0.295	0.321
	**Hosmer-Lemeshow chi-square**		6.936	0.544	16.890	0.31		28.508	000
	**Nagelkerke’s R-squared**		0.159		0.111			0.151	

## Data Availability

No additional data available.

## Appendix A

**Table A1:** The model’s descriptive statistics.

v	Model Variable	Minimum	Maximum	Mean	St. dev.	Asymmetry		Kurtosis	
		Statistic	Statistic	Statistic	Statistic	Statistic	Standard Error	Statistic	Standard Error
**QD13 (attitude towards robot use health—dichotomous)**	**Trust**	0	1	0.171	0.376	1.74	0.015	1.04	0.030
d15a (occupation)	**Occupation**	1	18	8.12	5.50	0.393	0.015	−1.41	0.030
d10 (gender)	**Gender**	1	2	1.55	0.498	−0.199	0.015	−1.96	0.030
d8r2 (education)	**Educational level**	1	8	2.44	1.04	1.89	0.015	8.07	0.030
d25 (community type)	**Type of community where he/she lives**	1	8	1.95	0.787	0.292	0.015	0.270	0.030
d40a (household composit.)	**Family situation**	1	20	2.19	1.06	2.18	0.015	17.78	0.030
d63 (social class)	**Social class**	1	9	2.58	1.46	1.94	0.015	6.39	0.030
d11r1 (age)	**Age**	1	4	3.08	1.00	−0.68	0.015	−0.803	0.030
Qd8 (experience)	**Experience of robot use**	0	2	0.128	0.366	2.90	0.015	8.15	0.030
qd9 (I read about robots)	**Information about robots**	1	3	1.54	0.521	0.078	0.015	−1.43	0.030
qd10 (attitude towards robots)	**Attitude towards robots**	1	5	2.51	1.03	0.93	0.015	0.397	0.030
qd11 (robot perception)	**Perception of robots**	1	5	3.34	0.914	−0.88	0.022	0.165	0.043
QD123 (ease of use new)	**Ease of use of robots**	1	5	4.34	0.968	−1.97	0.015	3.99	0.030
BENF2	**Fosters innovation**	−1.32	3.53	0.000	1.000	1.18	0.015	1.61	0.030
BENF1	**Facilitates the performance of tasks**	−3.22	1.27	0.000	1.000	−0.92	0.015	0.470	0.030

## Appendix B

**Table A2:** Degree of fulfilment of the hypotheses.

Hypothesis	No Experience	Average Experience	Considerable Experience
H1. The individual’s perceived usefulness of robot use influences how he/she feels about having a medical operation performed by a robot	YES	YES	NO
H1.1. The perception that robots facilitate the performance of complex and dangerous tasks influences how people feel about having a medical operation performed by a robot	YES	YES	YES
H1.2. The perception that robots foster care innovation influences how people feel about having a medical operation performed by a robot	YES	YES	NO
H2. The individual’s perceived ease of use of robots influences how people feel about having a medical operation performed by a robot	YES	NO	NO
H3. The individual’s level of emotional relationship with robots influences how people feel about having a medical operation performed by a robot			
H3.1. The individual’s degree of knowledge of robots influences how people feel about having a medical operation performed by a robot	YES	YES	YES
H3.2. The individual’s perception of robots’ ability to perform his/her habitual work influences how people feel about having a medical operation performed by a robot	YES	YES	YES
H3.3. The individual’s attitude towards robots influences how people feel about having a medical operation performed by a robot	YES	YES	YES
H4. The individual’s sociodemographic characteristics influence how people feel about having a medical operation performed by a robot			
H4.1. The individual’s sociodemographic profile influences how people feel about having a medical operation performed by a robot	YES	YES	YES
H4.2. The individual’s place of residence influences how people feel about having a medical operation performed by a robot	NO	NO	NO
H5. The individual’s prior experience of robot use has an influence on his/her perception of and relationship with robots	NO	NO	NO

## Appendix C

**Table A3:** Degree of fulfilment of the hypotheses for the gender, age, and educational level variables.

	GENDER		AGE				EDUCATIONAL LEVEL		
Hypothesis	Male	Female	15 to 24	25 to 39	40 to 54	55 and over	≤15 Years of Education	16–19 Years of Education	≥20 Years of Education
H1. The individual’s perceived usefulness of robot use influences how he/she feels about having a medical operation performed by a robot									
H1.1. The perception that robots facilitate the performance of complex and dangerous tasks influences how people feel about having a medical operation performed by a robot	YES	YES	YES	YES	YES	YES	YES	YES	YES
H1.2. The perception that robots foster care innovation influences how people feel about having a medical operation performed by a robot	YES	YES	YES	YES	YES	YES	NO	YES	YES
H2. The individual’s perceived ease of use of robots influences how people feel about having a medical operation performed by a robot	YES	NO	NO	NO	NO	YES	YES	NO	NO
H3. The individual’s level of emotional relationship with robots influences how people feel about having a medical operation performed by a robot									
H3.1. The individual’s degree of knowledge of robots influences how people feel about having a medical operation performed by a robot	YES	YES	YES	YES	YES	YES	NO	YES	YES
H3.2. The individual’s perception of robots’ ability to perform his/her habitual work influences how people feel about having a medical operation performed by a robot	YES	YES	YES	YES	YES	YES	YES	YES	YES
H3.3. The individual’s attitude towards robots influences how people feel about having a medical operation performed by a robot	YES	YES	YES	YES	YES	YES	NO	YES	YES
H5. The individual’s prior experience of robot use has an influence on his/her perception of and relationship with robots	YES	NO	NO	NO	YES	NO	NO	YES	NO
